# Technological Microbiology: Development and Applications

**DOI:** 10.3389/fmicb.2017.00827

**Published:** 2017-05-10

**Authors:** Luciana C. Vitorino, Layara A. Bessa

**Affiliations:** Laboratory of Agricultural Microbiology, Goiano Federal InstituteGoiás, Brazil

**Keywords:** biotechnology, food microbiology, biopolymers, plant growth-promoting microorganisms, environmental microbiology, biofactories

## Abstract

Over thousands of years, modernization could be predicted for the use of microorganisms in the production of foods and beverages. However, the current accelerated pace of new food production is due to the rapid incorporation of biotechnological techniques that allow the rapid identification of new molecules and microorganisms or even the genetic improvement of known species. At no other time in history have microorganisms been so present in areas such as agriculture and medicine, except as recognized villains. Currently, however, beneficial microorganisms such as plant growth promoters and phytopathogen controllers are required by various agricultural crops, and many species are being used as biofactories of important pharmacological molecules. The use of biofactories does not end there: microorganisms have been explored for the synthesis of diverse chemicals, fuel molecules, and industrial polymers, and strains environmentally important due to their biodecomposing or biosorption capacity have gained interest in research laboratories and in industrial activities. We call this new microbiology Technological Microbiology, and we believe that complex techniques, such as heterologous expression and metabolic engineering, can be increasingly incorporated into this applied science, allowing the generation of new and improved products and services.

## Introduction

The history of the use of biotechnological techniques by humanity is confounded by the history of the establishment of microbiology as a science. The first indication of the use of microorganisms for cereal grain fermentation to produce an alcoholic beverage was obtained from molecular evidence from the Neolithic village of Jiahu in China and dates to 7000 BC (McGovern et al., 2004). Similar evidence was found in the Zagros Mountains of northern Mesopotamia, dating to 5400–5000 BC (McGovern et al., 1996). The first indication of wine production comes from the presence of tartaric acid in an old jar, also dated to 5400–5000 BC, at the Neolithic site of Tepe in Mesopotamia (McGovern et al., 1996) and from grape juice residues, found in Dikili Tash in Greece and dated to 5000 BC (Valamoti et al., 2007). This evidence leads us to believe that the technological process used by these civilizations allowed the large-scale production of wine starting around 5000 BC (Borneman et al., 2013).

The Egyptians, who already used yeast to brew beer, began to employ this microorganism to make bread. Samples were found in different archeological sites dating to 2000–1200 BC (Samuel, 1996). The establishment and dissemination of fermentation practices throughout Asia, Mesopotamia, Egypt, and the Old World are traits of the empirical domestication of yeasts (Sicard and Legras, 2011), which later stimulated the interest of Louis Pasteur in explaining the true cause of fermentation (Pasteur, 1857). Evidence suggests that in 1856, Pasteur was approached by a beetroot-based alcohol producer from the Lille agricultural-industrial region, who faced production problems. Thus began the pioneering studies of Pasteur on lactic acid and alcoholic fermentations (Gal, 2008). He then became an admirer of the microscopic universe, describing the association of microorganisms with diseases and proposing vaccination methods such as used against anthrax (1881) and human rabies (1885) (Pasteur, 2002; Plotkin and Plotkin, 2011). Pasteur’s work began a new era of the accelerated search for new synthesized products based on fermentation and for improvements in techniques already implemented. His studies also provided support for the establishment of microbiology as a science, which had as its initial interest the sanitary control of diseases.

Technological Microbiology, however, started to draw the attention of the market when products originating from microbial activity began to be required on an industrial scale. This occurred with the glycerol demand for the manufacture of explosives during World War I (Wang et al., 2001) and the large-scale production of penicillin, discovered by Fleming, in the 1940s (Neushul, 1993).

The American economic expansion brought on by the end of World War II and known as the Golden Age of Capitalism (Stephen et al., 1991), as well as the knowledge of microbial genetics that was emerging at that time (e.g., Zinder and Lederberg, 1952; Jacob et al., 1960; Ames and Martin, 1964; Holloway, 1969; Sussman, 1970; Bagdasarian and Timmis, 1982), stimulated the emergence of microorganism-based industrial processes, triggering modern Technological Microbiology. However, Technological Microbiology is considered to have begun in the 1980s, following a decision made by the United States Supreme Court that allowed the patenting of a *Pseudomonas putida* variant that is effective in the organic digestion of compounds found in crude oil spills (Robinson and Medlock, 2005). The patent for a genetically modified microorganism, requested by Ananda Chakrabarty, contributed to a revolution in biotechnology that resulted in the issuance of thousands of patents, the founding of hundreds of new companies, and the development of thousands of bioengineering and food plants (Holloway, 2014).

The studies of Warner Arber, Hamilton Smith, and Daniel Nathans on bacterial endonucleases that hydrolyze the DNA of the viruses invading these microorganisms (Smith and Nathans, 1973; Arber, 1974) in the early 1970s earned these researchers the Nobel Prize in Physiology or Medicine in 1978. These enzymes, also known as restriction enzymes for “breaking” the DNA and providing gene fragments, became frequently used in biotechnological processes such as cloning, hybridization, fingerprinting, gene identification, and other genetic manipulations for the production of transgenic animals and plants.

The genetic modification of *Escherichia coli* in the 1970s allowed the production of artificial insulin, which was the first product obtained from recombinant DNA technology (Walsh, 2012) and was approved by the United States Food and Drug Administration in 1982 (Johnson, 1983). Over time, the selection of improved microbial strains became frequent, as did the manipulation of other microorganisms to obtain products to meet human demands, and as a result, Technological Microbiology has become a science essentially applied to several branches of production, including food, chemical, agricultural, and pharmacological.

Alternatively, the success of the genetic transformation carried out by Herbert Boyer and Stanley Cohen in California from the construction of chimeric *E. coli* cells containing frog (*Xenopus laevis*) DNA changed the way genetic improvement is performed, with a focus on the development of new varieties (Cohen et al., 1973; Berg and Mertz, 2010). In 1976, a thermostable DNA polymerase was isolated from the bacterium *Thermus aquaticus*. Kary Mullis and others contributors found that this enzyme could be used in the polymerase chain reaction (PCR) to amplify DNA fragments (Chien et al., 1976; Saiki et al., 1988). The development of that technique, as well as the enhancement of molecular cloning techniques using plasmids as vectors, expanded the possibilities of microorganism manipulation (Simon and Chopin, 1988; Olsen, 2016) and the large-scale production of microbial products, including those from modified microorganisms.

**FIGURE 1:**
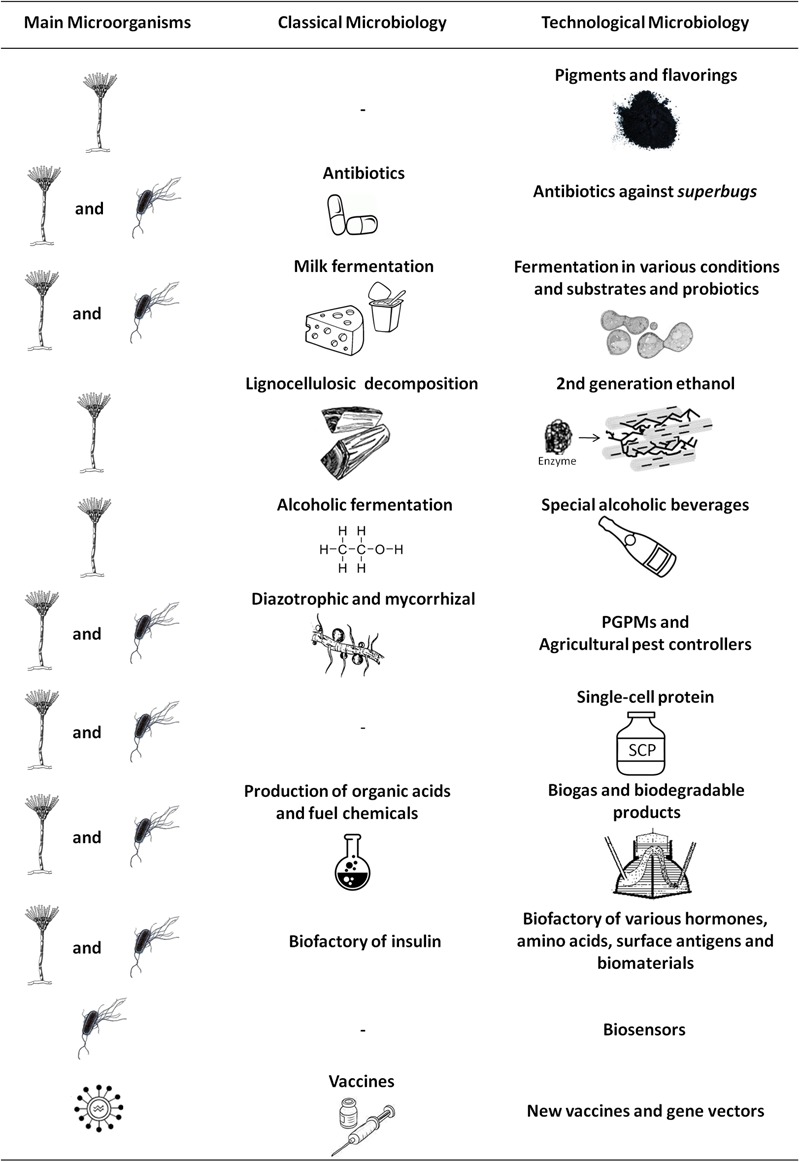
**The main applications of fungi, bacteria, and viruses for obtaining new or improved products.** Comparison between the possibilities generated by Classical Microbiology and Technological Microbiology, where the incorporation of techniques has led to market novelties, as well as to the improvement of commonly used products and services. PGPMs, plant growth-promoting microorganisms.

In addition, in the 1970s, Carl Woese and colleagues used the 16S rRNA molecule, a universally conserved sequence, as a taxonomic marker and revealed that our ignorance about microbial diversity was enormous, capable of hiding the existence of a new prokaryotic domain, the Archaea (Woese and Fox, 1977; Woese et al., 1990). Recent genomic discoveries have shown that the tree of life seems to be even more complex, as the existence of two extraordinarily complex and poorly studied groups has been revealed: the bacterial group Candidate Phyla Radiation (CPR) and the Archaea superphylum DPANN (Spang and Ettema, 2016). CPR bacteria have small genomes and unusual ribosomal compositions, in addition to lacking numerous biosynthetic pathways (Brown et al., 2015), whereas DPANN has been defined as a function of the metabolic capacity (Rinke et al., 2013; Castelle et al., 2015). These studies indicate that the tree of life may continue to grow in the future. Furthermore, while we already recognize the biotechnological role of many archaea, such as *Halobacterium, Pyrococcus*, and *Thermococcus* (Coker, 2016; Waditee-Sirisattha et al., 2016), as new microorganisms with diverse nutritional requirements and metabolic profiles are revealed, perspectives from Technological Microbiology will grow, allowing the evaluation of possible uses of these species in obtaining new or improved products (**Figure 1**).

Therefore, we have seen a contemporary improvement in Classical Microbiology through the discovery of new species, selection and improvement of known strains until the introduction of non-native genes for the acquisition of expressed products or new functional traits. We have decided to call this complex and applied microbiology Technological Microbiology, and although many of its fields overlap, to facilitate our discussion, we chose to divide it into six areas: Food Technological Microbiology, Agricultural Technological Microbiology, Chemical and Fuel Technological Microbiology, Environmental Technological Microbiology, Medical Technological Microbiology, and Materials Technological Microbiology, as follows.

## Food Technological Microbiology

Despite the application of biotechnological techniques to the food-processing industry and the agroindustry, which occurred prior to the technological advances of the 1970s, the current trend incorporates the use of genetically modified microorganisms or even the use of enzymes, dyes, and other compounds obtained from microbial metabolism with the aim of improving productivity, enhancing organoleptic characteristics, or even attributing new nutritional functions to certain foods. Microorganisms, therefore, may have two different roles in current food production. First, acting as starters in fermentations (in this case, GMOs or engineered microorganisms are not allowed). Secondly, they are used as factories for the production of food ingredients. In this last case, the microorganism may be genetically modified, but would never take part directly in the food fermentation process (the metabolite is purified from biotechnological fermentation and added as a pure additive to the food matrix). The participation of the microorganisms in this case is indirect.

Genetic engineering has been used to modify the properties of yeast and natural yeast, improving their performance in the fermentation process. In the future, breads and pastas of the better quality can be obtained in less time. Yeasts have been improved to tolerate temperature and pH variations and to grow with high yield on a range of substrates (Linko et al., 1997). Compounds involved in stress tolerance in yeasts, such as proline and trehalose, are promising for the development of resistant strains (Takagi and Shima, 2015). Thus, yeasts subjected to new processes such as UV radiation have allowed foods with new nutritional attributes to be developed, such as foods with increased vitamin D levels (Degré et al., 2008; Lipkie et al., 2016).

Selection of β-lyase-producing yeasts improves aromatic thiol release and, consequently, the sensory properties of wines (Belda et al., 2016), whereas the selection of yeasts specializing in certain processes such as flocculation may improve the fermentation of special wines such as sparkling wines (Tofalo et al., 2016). In turn, the current trend of using non-*Saccharomyces* strains, which in the past were considered yeasts of secondary importance or yeasts that produced undesirable changes, has positively impacted the vinification process, given the ability of the strains to produce enzymes, secondary metabolites, glycerol, ethanol, and other compounds that can increase the organoleptic complexity of wines (Padilla et al., 2016).

The prospection of lactic acid bacteria present in products fermented with various cultures (e.g., Satish et al., 2013; Mokoena et al., 2016) has created new sources of probiotics and the discovery of strains that can improve the quality of fermented products. Probiotics are living microorganisms that have been linked to host health benefits (Gawkowski and Chikindas, 2016). Currently, the best-known probiotic microorganisms are those belonging to the genera *Lactobacillus* and *Bifidobacterium* (Prasad et al., 2000). Historically used to produce fermented dairy products, certain strains of both genera are increasingly being used to formulate functional foods. The result of this phenomenon is the increase in the number of probiotic foods available on the market, including a rapidly emerging variety of non-dairy probiotic beverages (Enujiugha and Badejo, 2017).

A number of enzyme preparations of microbial origin have been evaluated in food processing. Amylases obtained from cultures of *Aspergillus niger* (e.g., Omemu et al., 2005; Djekrif-Dakhmouche et al., 2006; Adejuwon et al., 2015) or *Bacillus subtilis* (e.g., Ploss et al., 2016; Salman et al., 2016), for example, have been used in place of chemical additives in the treatment of wheat flour (e.g., Bueno et al., 2016), improving the preparation of dough for baking and allowing the acquisition of pre-cooked foods. *A. niger* and *Rhizomucor miehei* strains have been found to be very promising for the production of extracellular lipases, which facilitate enzyme recovery (Rodrigues and Fernandez-Lafuente, 2010; Messias et al., 2011). These microbial lipases are being employed in the hydrolysis of milk fat, improving the aromatization of dairy products. They can also enhance the aroma of beverages and the quality of margarine and mayonnaise (Sharma et al., 2001). Cellulases and pectinases, used especially in juice clarification and viscosity reduction, have also been easily recovered from cultures of filamentous fungi that are efficient in the degradation of plant biomass, such as *Cladosporium sphaerospermum, Penicillium chrysogenum* (Andersen et al., 2016), and *Trichoderma viride* (Ismail et al., 2016). In addition, the technology for immobilization of these enzymes in prefabricated supports or polymer matrices improves their stability, activity, and selectivity, favoring their application and reuse for long periods in industrial reactors (Mateo et al., 2007; Sheldon, 2007).

In addition, microbial enzymes have been used to obtain natural scents and flavorings for foods (Carroll et al., 2016), although these compounds can often be directly obtained from the general metabolism of filamentous fungi, such as *A. niger* and *Pycnoporus cinnabarinus*, which can act together in a process that leads to the synthesis of vanillin, an important food flavoring, from autoclaved maize bran (Lesage-Meessen et al., 2002). Additionally, yeasts of the genus *Pichia* have been added to coffee fermentation to improve the quality and flavor of the beverage because they increase the production of the natural flavoring isoamyl acetate (Saerens and Swiegers, 2016). Microbial biosynthetic pathways have been explored mainly because they can enzymatically convert inexpensive precursors, such as glucose or glycerol, into expensive aromatic compounds. One example is the synthesis by *E. coli* of acetoin, which is responsible in part for a buttery aroma, using glucose as a substrate (Nielsen et al., 2010).

High rates of population growth have increased the demand for new foods around the world. Protein extracted from cultivated microbial biomass (single-cell protein – SCP) can be used for protein supplementation in basic diets, replacing expensive conventional sources and alleviating the problem of protein shortages (Anupama and Ravindra, 2000). SCP has been widely used as a source of protein in animal and human food (Adedayo et al., 2011). Many bacterial strains of *Bacillus, Hydrogenomonas, Methanomonas, Methylomonas*, and *Pseudomonas* have been used as substrate for the production of S on an industrial scale because these bacteria can contain approximately 80% crude protein in the total dry weight. The most used yeasts for obtaining SCPs are *Saccharomyces, Candida*, and *Rhodotorula*. Cultivation of yeasts is more practical because these microorganisms are able to use a wide variety of substrates (Patelski et al., 2015); however, the SCPs obtained are insufficient in sulfur-containing amino acids. The most commonly used filamentous fungi are *Fusarium, Aspergillus*, and *Penicillium*, and among the prokaryotic algae, the most used belong to the genus *Spirulina*, with approximately 65% of their dry weight consisting of protein (Nalage et al., 2016). The possibility of using microorganisms to obtain food, food additives, or even microbial biomass for food has reinvigorated the food-processing industry, which sees new possibilities for conventional foods, such as flavors, textures, and aromas, or even the discovery of new foods.

## Agricultural Technological Microbiology

Recently, the interest in microorganisms has focused on compounds with pesticidal activity, mainly herbicidal, insecticidal, and nematicidal. The first commercially registered mycoherbicide consisted of a suspension of chlamydospores of *Phytophthora palmivora* to control *Morrenia odorata* (McRae, 1988), and since then, many other plant parasite and phytotoxin-producing microbial species have been identified. *Colletotrichum gloeosporioides* (Penz) Sacc. f. sp. *aeschynomene* can induce symptoms of anthracnose in *Aeschynomene virginica*, thus controlling this legume, which is a rice and soybean weed. On the other hand, *Puccinia canaliculata* can control yellow nutsedge (*Cyperus esculentus* L) by completely inhibiting flowering and reducing tuber formation (Duke et al., 2015). Bioherbicides, however, have not been widely applied in agronomic and horticultural crops for weed management because they have a number of requirements, such as ideal humidity conditions, which diminish their effectiveness when compared to chemical herbicides. In the future, biotechnological advances will likely reverse this situation and improve the performance of bioherbicides.

The endotoxin proteins Cry and Cyt are currently best known as pesticides. These endotoxins are synthesized by the soil bacterium *B. thuringiensis* (Bt) and have an entomopathogenic action, controlling the pests present in cabbage, potato, and grains (Sarwar, 2015a). Several transgenic species expressing Bt protein crystals, such as tomato, tobacco, and corn, have been cultivated worldwide because they have been successful in preventing the spread of caterpillars, especially Lepidoptera (Khan et al., 2016). Caterpillars and eggs of pests such as *Spodoptera frugiperda* can also be infected by baculovirus, thus reducing the agricultural losses caused by this caterpillar, especially in corn. In addition, the progress achieved by the genetic improvement of this virus has increased its effectiveness as an insecticide (Popham et al., 2016). Several fungi pathogenic of insects are also being used as control agents, including *Beauveria, Metarhizium*, and *Paecilomyces*. These are most frequently used against leaf caterpillars in greenhouses or other places where the humidity is relatively high (Sarwar, 2015b).

In recent years, much progress has been achieved in the development and commercialization of bionematicides (Wilson and Jackson, 2013). Examples of this are the products of the bacterium *Streptomyces avermitilis*, which are metabolites known as avermectins. These are model pesticides, as they are non-toxic to mammals and active against nematodes, even at very low doses. Thus, filtrates of *B. firmus* cultures induce paralysis and mortality of adult nematodes and larvae, including *Radopholus similis, Meloidogyne incognita*, and *Ditylenchus dipsaci*, which suggests that the synthesis of toxic metabolites (Mendoza et al., 2008) is involved in the control of these pests. Toxic metabolites are also produced by *Myrothecium verrucaria* when grown in bioreactors, and when in contact with adult nematodes, the metabolites in suspension kill the adults, in addition to inhibiting egg development and hatching (Twomey et al., 2000). By contrast, the endospores of the bacterium *Pasteuria* sp. use parasitism as a method of control. When these endospores come into contact with nematodes such as *Meloidogyne* spp., *Heterodera* spp., *Globodera* spp., and *Belonolaimus* spp., they germinate, become parasitic, and strongly decrease host fecundity (Davies et al., 2011).

Among the microorganisms that act in the biological control of pests, the most widely disseminated species are the fungi belonging to the genus *Trichoderma*. These fungi are saprophytes, mycoparasite decomposers, and plant symbionts, usually associated with soil ecosystems, and have a global geographical distribution (Druzhinina et al., 2011). This range of lifestyles within the genus explains why *Trichoderma* is the source of many strains commercially used in biological control (Howell, 2003). *Trichoderma* spp. parasitize and successfully control phytopathogenic fungal species such as *Sclerotinia* (Jones et al., 2014, 2016), *Fusarium* (Saravanakumar et al., 2016), *Verticillium* (Carrero-Carrón et al., 2016), and *Macrophomina* (Khaledi and Taheri, 2016), among others, and have nematicidal effect on the gall-forming *Meloidogyne* (Sahebani and Hadavi, 2008; Feyisa et al., 2016; Sokhandani et al., 2016). This functional characteristic of *Trichoderma* and other biocontrol species responds to the increasing call for practices that minimize the side effects left by pesticides, such as resistance in pest populations, reduction of soil and water quality, and the generation of residues with harmful effects on non-target organisms.

Sustainable agriculture, however, provides not only the control of phytopathogens but also the use of functional microbial characteristics related to the promotion of plant growth. Symbiotic microorganisms such as mycorrhizal fungi and rhizobacteria develop activities that can improve plant fitness, facilitating nutrient acquisition by the plant. Mycorrhizal fungi and roots are complementary in plant foraging within nutrient patches (Cheng et al., 2016) and facilitate the acquisition of phosphorus by the plant, through the expression of genes that code for inorganic transporters of this ion (Walder et al., 2016). Likewise, PGPRs (plant growth-promoting rhizobacteria) act through direct and indirect mechanisms to promote plant growth. Direct mechanisms include mainly biofertilization, with nitrogen synthesis by strains belonging to the genera *Rhizobium, Sinorhizobium, Mesorhizobium, Bradyrhizobium, Azorhizobium*, and *Allorhizobium*, and the stimulation of root growth through the synthesis of auxins, cytokinins, and gibberellins. Indirect mechanisms are related to the reduction of susceptibility to diseases, including antibiosis, induction of systemic resistance and competition for nutrients and niches (Lugtenberg and Kamilova, 2009).

On the other hand, endophytic microorganisms colonize plant tissues without triggering any disease symptoms, establishing a stable long-term interaction with the host plant. During the interaction, endophytes synthesize bioactive metabolites that may confer greater fitness to the plant. This promotion of growth by endophytic action may be a consequence of nitrogen fixation, synthesis of phytohormones, biocontrol of phytopathogens through the synthesis of antibiotics or siderophores, competition for nutrients, and the induction of systemic disease resistance (Ahmad et al., 2016). However, the bioprospection and characterization of these microorganisms, associated with the most diverse plant species, is aimed not only at obtaining strains of agronomic importance but also at the identification of species that produce metabolites with potential for the synthesis of antibiotics (e.g., Ma et al., 2016), as well as potential for obtaining biotechnologically important chemicals.

## Chemical and Fuel Technological Microbiology

Obtaining chemicals such as organic acids via microbial activity is very promising, especially if it is thought to occur from renewable carbon sources. Most organic acids are natural products or intermediates of the microbial metabolism present in important metabolic pathways (Sauer et al., 2008). Due to their functional groups, these acids, such as acetic, citric, lactic, and succinic acid, are extremely useful as raw materials for the chemical or food industry. Citric acid, for example, has been required on the market for use as a food additive, and all annual worldwide industrial-scale production occurs via the fermentation of glucose, beet molasses, cane molasses, or corn starch using *A. niger* (Adham, 2002; Ikram-ul et al., 2004; Wang et al., 2016). On the other hand, all the annual world production of lactic acid also comes from the fermentation performed by microorganisms. This acid and its derivatives are widely used in the food, pharmaceutical, leather, and textile industries. In addition, lactic acid fermentation processes have recently received more attention because of the growing demand for new biomaterials, such as biodegradable products and biocompatible polylactics (Gao et al., 2011). The most common method for obtaining this acid is using *Lactobacillus* spp. cultivated in whey (Hofvendahl and Hahn-Hägerdal, 2000). However, it may also be obtained via the activity of *Rhizopus* sp. under aerobic conditions in glucose-rich medium and with limited amounts of nitrogen (Papagianni, 2004; Fu et al., 2014), and even via the fermentation of *Saccharomyces cerevisiae* in glucose- and cane juice-based medium (Saitoh et al., 2005; Valli et al., 2006). In the future, the microbiological processes for obtaining a variety of organic acids are expected to will be competitive, become established in the market, and allow for an annual increase in the production of these compounds.

The microbial production of acetone and butanol, efficiently performed by the genus *Clostridium*, was one of the first large-scale industrial fermentation processes to gain global importance, but this production has been losing ground to chemical synthesis. Similarly, the centennial microbial synthesis of glycerol was impacted by the inability to compete with chemical synthesis from petrochemical feedstocks. However, in a scenario where the cost of propylene increased because its availability decreased, especially in developing countries, glycerol became an important raw material for the production of various chemicals, which made its alternative synthesis by fermentation more attractive (Wang et al., 2001).

Likewise, the production of 1,3-propanediol (1,3-PDO), which occurs through the fermentation of glycerol by bacteria of the genus *Clostridium* or *Enterobacteriaceae*, a technique described in 1881, lost status front to chemical synthesis through petroleum products, remaining forgotten for more than a century. In the last decade, however, research related to the synthesis of microbial 1,3-PDO expanded considerably (Biebl et al., 1999) because this diol began to be used in the synthesis of biodegradable polymers and for obtaining solvents, films, adhesives, antifreezes, and polyesters. Currently, a potentially viable alternative for the synthesis of 1,3-PDO is the use of genetically modified microorganisms. Genes from pathogenic bacteria, such as *Citrobacter freundii* and *Klebsiella pneumoniae*, were recently introduced in *E. coli* to allow the efficient synthesis of 1,3-PDO from waste glycerol (Przystałowska et al., 2015).

This synthesis of chemicals through microbial metabolic processes meets an urgent need to reduce dependence on fossil fuels for energy generation. In modern biorefineries, renewable resources such as biomass or waste products are converted into substrates susceptible to microbial action (Sauer, 2016), and thus, interest in bio-based chemicals has recently been renewed because increasing climate change and environmental problems have pushed the industry, moving it away from fossil fuel consumption and toward renewable raw materials (Moon et al., 2016). Microorganisms have also been potentially explored for the production of a new generation of biofuels (Liao et al., 2016). The production of second-generation ethanol, for example, obtained from lignocellulosic biomass, already occurs in some countries, although improvements are still needed to make the technology economically competitive. Recent developments such as the discovery of functional xylose isomerases (Kuyper et al., 2005) resulted in the creation of new yeasts capable of fermenting 5-carbon (C5) sugars, as well as 6-carbon (C6) sugars. Co-fermentation of C5 sugars with cane juice can produce up to 37% more ethanol in first-generation fermenters (Losordo et al., 2016). Another problem to be overcome for the effective production of second-generation ethanol is the tolerance to acetic acid. This acid is one of the main inhibitors of lignocellulose hydrolysates. The polygenic basis of the high acetic acid tolerance present in some strains of *S. cerevisiae* is still unknown, but its identification may lead to greater efficacy in improving acetic acid tolerance in strains without negatively affecting other industrially important yeast properties (Meijnen et al., 2016). However, in addition to yeast genetic improvements, the prospection of new cellulose sources, such as forestry and crop residues (eucalyptus bark, corn, and rice husks), and the development of pretreatment techniques (e.g., McIntosh et al., 2016) can leverage the production of second-generation ethanol.

In addition to bioethanol, other energy molecules such as biogas can be obtained from the microbial conversion of biomass. Biogas is a combination of methane, CO_2_, nitrogen, H_2_S, and traces of other gasses produced by anaerobic digestion (AD) (Appels et al., 2008). Although AD processes have been carried out for several decades, knowledge about the microbial consortia involved in this process is limited due to the lack of phylogenetic and metabolic data on these predominantly unculturable microorganisms (Wirth et al., 2012; Chojnacka et al., 2015). Studies carried out to isolate and identify the microbial community associated with the production of biogas revealed the presence of *Proteobacteria, Chloroflexi, Firmicutes, Bacteroidetes, Actinobacteria, Bacteroides, Acidobacteria*, and *Spirochetes* (Chouari et al., 2005; Chojnacka et al., 2015). Methanogenic Archaea, such as *Methanosarcina barkeri, M. frisius*, and *Methanobacterium formicicum*, were also identified in anaerobic digestions (Godon et al., 1997; Satpathy et al., 2016). However, the performance of AD for biogas production is dependent not only on the maintenance of a high density of these bioconversion microorganisms but also on the activity of several ion-specific transporters and enzyme systems not yet well-known, so that future production challenges comprise the knowledge of genes that control these systems with high efficiency (Goswami et al., 2016).

**FIGURE 2:**
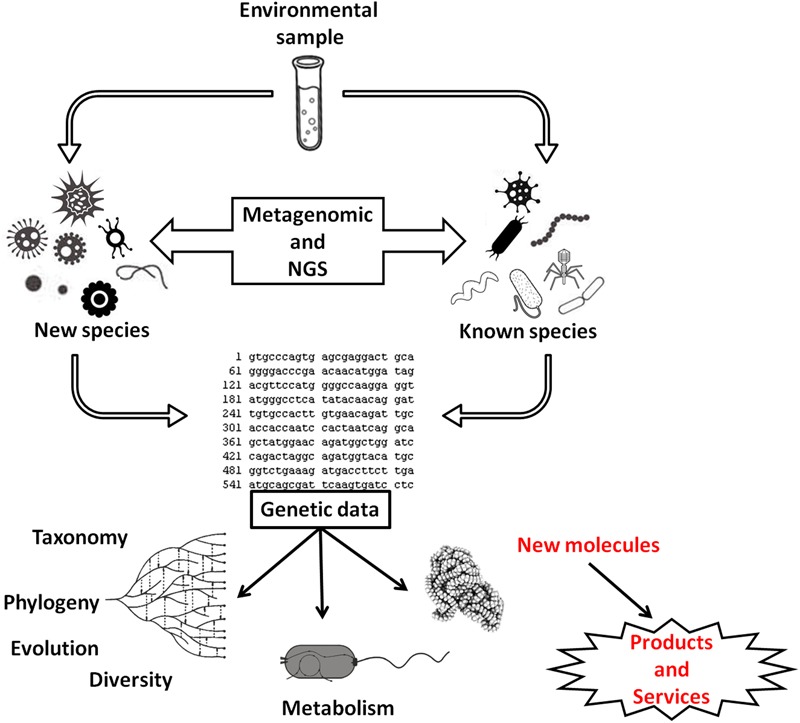
**Use of metagenomics and next-generation sequencing in the study of microbial communities to obtain genetic data.** These data can be used in studies of microbial diversity, group phylogeny, species diversification, and microbial metabolism. These data have also allowed the discovery of new or modified molecules used to obtain improved products, new products, or services.

The expectation is that, in the future, at least 25% of all bioenergy can originate from biogas (Holm-Nielsen et al., 2009), and therefore, studies that seek to optimize the methanogenesis process or describe the structure of microbial communities have been encouraged (e.g., Ennouri et al., 2016; Mulat et al., 2016; Suksong et al., 2016). Metagenomic approaches associated with next-generation sequencing (NGS) techniques will help to unravel the diversity of natural communities and in biogas fermenters communities (e.g., Schlüter et al., 2008). However, studies have shown that most of the microorganisms isolated from the reactors are still unexplored (e.g., Krause et al., 2008; **Figure 2**) and may be a source for new products and services in the future.

## Environmental Technological Microbiology

A large variety of microorganisms, including heterotrophic or autotrophic aerobic bacteria, actinomycetes, fecal coliforms, and thermophiles, as well as yeasts and other fungi, have been reported in solid waste composting processes (Beffa et al., 1996; Tiquia et al., 2002). Many factors determine the microbial community present during composting, but under aerobic conditions, temperature is the main factor determining not only the microbial types but also the species diversity and metabolic rate (Hassen et al., 2001). On the other hand, the direct use of microbial enzymes in the treatment of effluents, especially industrial effluents, has been encouraged because the enzymatic action is faster, dispensing with the conditions necessary for the fermentative process. Lipases, for example, are used in the treatment of wastewater containing mainly triglycerides (Jamie et al., 2016). The presence of these enzymes in activated sludge and in other aerobic degradation processes is important for the continuous removal of the fat layers formed on the surface of aerated tanks to allow oxygen transport (Hasan et al., 2006). Peroxidases, phenoloxidases, dioxygenases, and phenoloxidase-like compounds have also been used for the removal of contaminants present in wastewater (Durán and Esposito, 2000). Peroxidases, polyphenol oxidases, and tyrosinases obtained from microorganisms such as *P. syringae, Arthromyces ramosus*, and *Agaricus bisporus* may be applied to the removal of phenols, biphenols, and chlorophenols (Tatsumi et al., 1996; Tong et al., 1998; Akay et al., 2002; Kampmann et al., 2014). Laccases of *P. cinnabarinus* were found to be efficient for the degradation of benzopyrene (Rama et al., 1998), while manganese peroxidases of *Phanerochaete chrysosporium, Nematoloma frowardii*, and *Phlebia radiata* can be applied to the elimination of lignin in wastewater (Hofrichter et al., 1999; Kunz et al., 2001).

Currently, research efforts have focused on integrating the treatment of solid wastes or even wastewater with the use of microbial fuel cells (MFC), i.e., microbial cells that use electrons donated by low-value organic substrates, contained in the waste, to generate energy (Xu et al., 2016). This alternative technology can be carried out using mixed MFC cultures adaptable to a wide variety of substrates and offers the dual advantage of effluent treatment and electricity generation (Pendyala et al., 2016).

Research efforts have also been directed at improving the purification of drinking water. A recent biotechnological process called biologically active carbon (BAC) has been found to be very efficient in removing water contaminants. In this process, microbial cells colonize the surface of the granular activated carbon (GAC) used in the filtering mechanism. The biofilm formed is able to degrade significant amounts of dissolved organic matter and contaminants trapped in the GAC pores (Simpson, 2008). In addition, the BAC biofilm can also biodegrade the cyanotoxins and organic substances that can change the taste and odor of potable water (Brown and Lauderdale, 2006).

Waste treatment based on enzymatic processes tends to be less expensive; however, the enzymes are biodegradable, and further studies and prospection of microbial enzymes that are thermostable or resilient to large pH variations are needed. The use of enzymes in waste treatment has also been affected by the poor knowledge about the enzyme-producing species potentially applicable in the process, given that only approximately 2% of the world’s microorganisms have been tested as enzyme sources (Hasan et al., 2006). Genetic improvement, as well as the genetic manipulation of cells and the heterologous expression of genes, is expected to help increase the enzymatic biosynthesis in microorganisms of interest, or even to contribute to the development of microorganism biofactories for important enzymes not only for food or industry but also for environmental applications, thereby expanding alternatives for the elimination of the wastes that have historically accumulated in soils and watercourses.

The increasing market demand for biodegradable polymers has also stimulated the prospection of microorganisms that act in the synthesis of these compounds. The class of biodegradable biopolymers of major interest is the polyhydroxyalkanoates (PHAs), and the best known among these are poly(beta-hydroxybutyrate; PHB), poly(beta-hydroxyvalerate; PHV), and poly(hydroxybutyrate-co-valerate; PHB-V), the latter being commercially known as Biopol. These biopolymers are accumulated intracellularly by bacteria as a carbon and/or energy reserve under the limitation of a nutrient essential for their growth, such as nitrogen, phosphorus, sulfur, or oxygen (Philip et al., 2007). The species *Cupriavidus necator* is one of those responding more favorably to the conditions for industrial production. This bacterium can accumulate approximately 80% of its dry mass in polymer and uses different types of substrates, such as glucose, fructose, and crude glycerin (Figueiredo et al., 2014). Biopolymers offer a possible solution for eliminating the problem of the residuals associated with petroleum-based plastic, but for researchers, the real challenge lies not in obtaining these molecules, which are the products of microbial metabolism, but in finding applications that consume large amounts of these materials, promoting price reductions and allowing biopolymers to compete economically in the market (Mohanty et al., 2000).

Another aspect of environmental technological microbiology is advances in the knowledge and use of the symbiotic relationship between plants and mycorrhizal fungi as a strategy to increase plant biomass or increase the yields of products of agricultural or pharmacological interest (e.g., Boyer et al., 2016; Gabriele et al., 2016; Köhl et al., 2016). Such benefits are the product of the positive and multifunctional roles of mycorrhizal fungi in plant nutrition, pathogen protection, stress tolerance, and soil structure supply (Smith and Read, 2008). Due to their unique biological traits, which include obligatory biotrophy and intracellular development within plant tissues, and multiple ecological functions, arbuscular mycorrhizal fungi (AMFs; which are also thought to have helped plants conquer terrestrial environments) have aroused agronomic interests with the aim of sustainable production with low chemical consumption (Lanfranco et al., 2016). In sustainable agriculture, AMFs are known as biofertilizers. In the plant-microorganism relationship, the mineral nutrients (mainly phosphorus, nitrogen and water) are extracted from the soil through the extensive network of hyphae and transferred to the plant, and organic compounds of carbon are transferred from the plant to the AMF. These microorganisms therefore reduce the need for application of chemical fertilizers in soils (e.g., Abdel-Fattah et al., 2016; Oliveira et al., 2016a,b). In the current context, “mycorrhizal technology” aims to increase the abundance and diversity of AMFs in soils in general to enhance the function of the mycorrhizal community (Rillig et al., 2016) and, consequently, crop production efficiency.

## Medical Technological Microbiology

The participation of microorganisms in the generation of medical products or services involves four distinct aspects: (1) biocontrol of diseases, (2) production of vaccines, (3) production of antibiotics, and (4) production of biotherapeutics (hormones, biomaterials, and others). These aspects will be discussed throughout this session.

**FIGURE 3:**
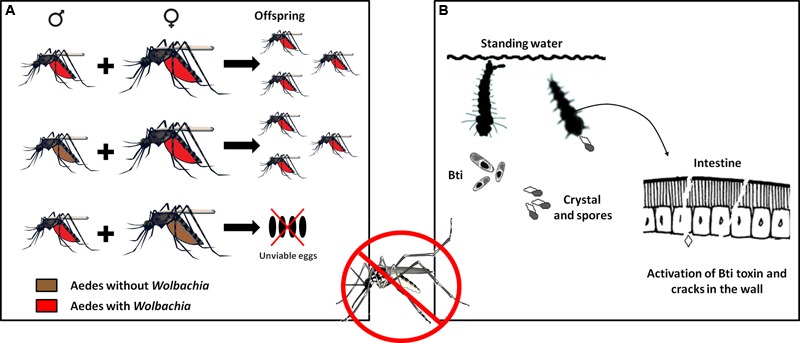
**Use of Technological Microbiology to prevent the proliferation of the *Aedes aegypti* vector and the DENV virus. (A)** Mechanisms of transmission of the bacterium *Wolbachia* to the offspring of the vector. Cytoplasmic incompatibility causes females with *Wolbachia* to always breed offspring with *Wolbachia*, whether mating with males with or without the bacterium. When females without *Wolbachia* mate with males with *Wolbachia*, the fertilized eggs die. With successive generations, the number of male and female mosquitoes with the bacterium tends to increase until the entire mosquito population bears this characteristic. **(B)** Use of *Bacillus thuringiensis israelensis* bioinsecticide to fight dengue. This bacterium synthesizes protein crystals that, when consumed by *Aedes* larvae, solubilize in the mosquito’s intestine and are transformed into efficient toxins that damage the intestinal wall, allowing the attack of pathogenic bacteria that cause the death of the larva.

A problem commonly encountered in developing countries is the difficulty of implementing public policies to control the spread of parasitic vectors such as those of the genera *Aedes* and *Anopheles*. However, recent epidemic outbreaks of emerging and reemerging diseases have stimulated the development of biotechnological techniques that can not only assist diagnosis but also serve as alternatives for controlling transmission. An example is the potential presented by the introduction of the bacterium *Wolbachia* as an endosymbiont of the mosquito *Aedes aegypti*, which transmits diseases such as dengue, yellow fever, chikungunya, and the more recently detected Zika virus (Walker et al., 2011). The focus of this approach is on the reduction of mosquito longevity and not on abundance. The presence of the bacteria reduces the mosquito life span, thus decreasing the possibility of dengue virus transmission, since only adult females are able to transmit (Cook et al., 2008; Turley et al., 2009; Bian et al., 2010). Mosquitoes containing the *Wolbachia* wMelPop-CLA strain showed an approximately 50% reduction of the survival of females compared to mosquitoes without the strain (McMeniman et al., 2009). These bacteria are transmitted vertically from the female to the offspring. To guarantee this transmission, the bacterium manipulates its host in diverse ways such as feminization, death of males, parthenogenesis, and cytoplasmic incompatibility. In cytoplasmic incompatibility, fertilization of females not infected with *Wolbachia* by infected males results in embryonic mortality. By contrast, females infected with the bacterium will produce the highest number of viable offspring, increasing the number of infected individuals in the population (**Figure 3A**). Cytoplasmic incompatibility facilitates the propagation of *Wolbachia* in natural populations and their persistence over time (McMeniman et al., 2009).

Another alternative, also focused on preventing the proliferation of the dengue virus vector, is the use of a biological insecticide. The action of this insecticide is based on the activation of endotoxins (Cry and Cyt) produced naturally by the bacterium *B. thuringiensis* serotype *israelensis* (Bti) (**Figure 3B**). These toxins, previously mentioned in this text, are inactive crystals that, when ingested by *Aedes* larvae, are solubilized by intestinal proteases and converted into active toxins that interact with the cell membrane of the midgut, leading to pore formation, cell lysis, septicemia, and finally death (Gill et al., 1992; de Maagd et al., 2001). This alternative has been successfully applied in several regions of the world (e.g., Mohiddin et al., 2016; Setha et al., 2016), and many Bti-based commercial products have been introduced to the market, although some strains of *A. aegypti* have already shown resistance to Bt toxins (Paris et al., 2011, 2012; Wu et al., 2016).

At the same time, the technological race for the development of a vaccine against dengue continues. Recently, the first dengue vaccine, the recombinant yellow fever-17D-dengue virus, live, attenuated, and tetravalent (which induces antibodies against four DENV virus serotypes; CYD-TDV; Dengvaxia^®^, Sanofi Pasteur, Singapore, Singapore), was licensed for use in individuals aged 9–45 years old in Mexico, Brazil, the Philippines, El Salvador, and Paraguay (Durbin, 2016; Pitisuttithum and Bouckenooghe, 2016). This represents an important complement to the other techniques that focus mainly on vector control. This vaccine, however, is currently only available under private health care systems at very high prices.

The HPV (human papilloma virus) vaccine is another example of a newly developed vaccine that has been widely used in many countries. The focus of campaigns in developing countries has been on women who are not yet sexually active, although being sexually active does not contraindicate the vaccine, which can be tetravalent (protects against types 6, 11, 16, and 18) or bivalent (protects against types 16 and 18). This virus has been frequently linked to cases of cervical cancer, with types 16 and 18 accounting for approximately 70% of cases (Cutts et al., 2007). However, these same viral types account for 86–95% of cases of non-cervical cancer, i.e., anal, oropharynx, vulval, and vaginal cancers in women, as well as anal, oropharyngeal, and penile cancers in men (Gillison et al., 2008). Thus, prophylaxis should also extend to men as a way to prevent potential non-cervical cancers; however, affordable prices, funding mechanisms, and multidisciplinary partnerships are essential for the HPV vaccine to reach most populations in need, especially considering that cervical cancer is the second leading cause of death of cancer in women and is more worrying in populations that do not have screening programs to detect precursor lesions (Roden and Wu, 2006).

**FIGURE 4:**
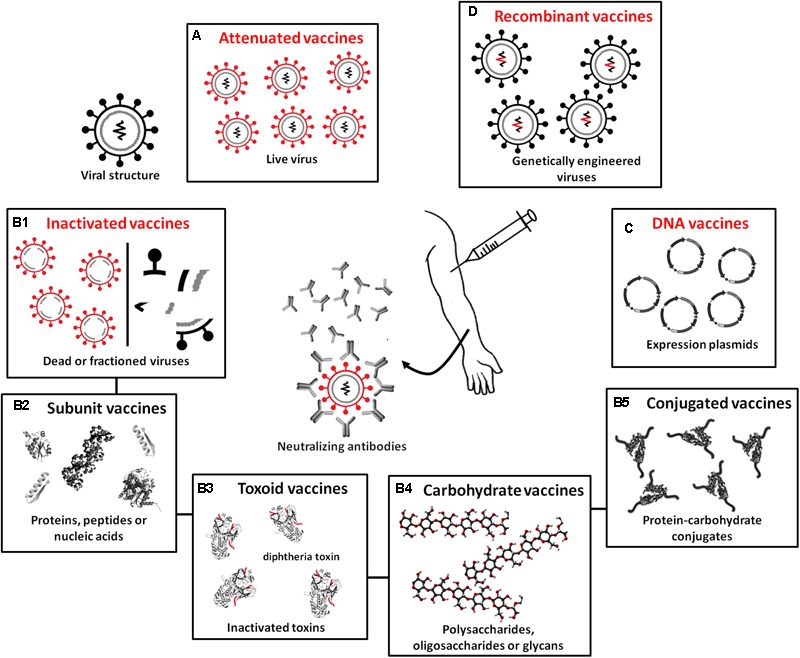
**Different biotechnological techniques used in the production of currently available vaccine types. (A)** Attenuated or live vaccines, which use attenuated pathogens. **(B)** Inactivated vaccines containing completely inactivated or fractionated pathogens or only antigenic components of these pathogens, subdivided into **(B1)** whole or fractioned; **(B2)** subunit vaccines, which use proteins, peptides, or nucleic acids as antigens; **(B3)** toxoids, which use inactivated pathogen toxins as antigens; **(B4)** carbohydrate vaccines produced from polysaccharides, oligosaccharides, and glycans; and **(B5)** conjugate vaccines, which have polysaccharides combined with transport proteins. **(C)** DNA vaccines carrying plasmids containing genes encoding immunogenic antigens. **(D)** Recombinant vaccines containing viruses engineered to carry genes encoding antigens from other disease-causing viruses.

For the production of vaccines, microorganisms do not function as biofactories but are instead only used (whole or fractionated) to stimulate the synthesis of specific antibodies. Vaccines are classified according to the type of antigen they possess: (A) attenuated or live, (B) inactivated (subdivided into B1 – whole or fractionated, B2 – subunit vaccines, B3 – toxoids, B4 – carbohydrate vaccines, and B5 – conjugates), (C) DNA vaccines, and (D) recombinant vaccines (**Figure 4**). In attenuated vaccines, the pathogens (virus or bacteria) are alive and induce immune reactions similar to those resulting from a real infection (Plotkin et al., 2008). Attenuated vaccines have been efficiently developed for a range of diseases: mumps, polio (Sabin), rubella, measles, smallpox, chickenpox, tuberculosis, yellow fever, and dengue. These vaccines are considered highly immunogenic and efficiently stimulate humoral immunity, such that only one dose is capable of conferring immunity for decades. Recent studies have revealed strategies for the development of attenuated strains of the influenza virus that trigger robust immune responses (e.g., Si et al., 2016; Wang et al., 2017). These studies not only provide perspectives for producing more effective vaccines against influenza but also suggest innovative approaches for the generation of live attenuated strains for viral pathogens, which remains a challenge (Bournazos and Ravetch, 2017). Studies have also shown that live vaccines induce non-specific immunity, conferring protection beyond the target pathogen (e.g., Aaby et al., 2010; Higgins et al., 2014; Sørup et al., 2014), and that revaccination may be indicated to reduce general mortality caused by all diseases (Benn et al., 2016).

Inactivated vaccines, which are classified as whole or fractionated, contain completely inactivated or fractionated pathogens or only antigenic components of these pathogens (including subunit vaccines, toxoid vaccines, carbohydrate vaccines, and conjugate vaccines). In general, these vaccines are effective stimulators of humoral immune responses, but in many cases, multiple doses are required for long-term immunity since they do not stimulate the production of immunoglobulin A (IgA) or cytotoxic T cell responses because the virus does not replicate. The advantage of inactivated vaccines is that they are safer because the virulence of dead organisms cannot be reversed. Inactivated vaccines are currently available for hepatitis A, rabies, cholera, influenza, poliomyelitis (Salk), typhoid fever, and pertussis. Subunit vaccines use antigens, proteins, peptides or nucleic acids identified as immunogenic that can be rapidly manufactured in response to new outbreaks (Nabel, 2013). These antigens are purified from microorganisms, produced by recombinant DNA techniques, or chemically synthesized. They are poorly reactogenic, which is an advantage in terms of adverse effects but a disadvantage in terms of stimulating potent and long-lasting immune responses (Bobbala and Hook, 2016). Thus, these vaccines often require the co-administration of efficient adjuvants to activate and modulate immune responses (Reed et al., 2009; Schmidt et al., 2016). Despite this poor reactogenicity, subunit vaccines have been developed against a variety of pathogens, including *Streptococcus pneumoniae*, hepatitis B virus, and HPV. By contrast, toxoid vaccines stimulate the immune response by using inactivated pathogen toxins as antigens. Vaccines of this type are available for tetanus, diphtheria, and anthrax. Carbohydrate vaccines are developed based on the knowledge that the vast majority of pathogens have dense distributions of polysaccharides, oligosaccharides, and complex glycans on their cell surface (glycocalyx) and that infected organisms detect the presence of pathogens through glycocalyx recognition and pattern recognition receptors (PRRs) that stimulate host defense responses (Astronomo and Burton, 2010; Pifferi et al., 2017). Low immunogenicity is a major obstacle to the manufacture of carbohydrate vaccines, and because of the lack of long-term immune protection by these antigens, few such vaccines are commercially available today. These include vaccines against *Salmonella typhi* and *Neisseria meningitides* (Keitel et al., 1994; King et al., 1996). Traditional covalent linkages between carbohydrates and carrier proteins have been used to increase immune responses to polysaccharide antigens (Nishat and Andreana, 2016), thereby obtaining conjugate vaccines. In this case, proteins function as carriers for capsular polysaccharides, which induce T-cell dependent antibody responses and B-cell differentiation in plasma cells and long-term memory. As a result, protein-polysaccharide vaccines induce immunological memory, a protection that is longer lasting than that induced by the use of simple polysaccharide antigens (Knuf et al., 2011; Frenck and Yeh, 2012; Pichichero, 2013). Conjugate vaccines are already currently used in the control of *Haemophilus influenza* type B and *S. pneumoniae*.

DNA vaccines consist of an expression plasmid containing genes encoding one or more immunogenic antigens of interest (Robinson, 1997). The use of viral promoters enhances gene expression and improves mRNA stability related to antigen synthesis. In addition, the incorporation of immunological adjuvants and new methods of insertion of this material into cells of the host organism beyond the intramuscular and dermal routes have increased the popularity as well as the immunogenicity of DNA vaccines, since the action of nucleases can inactivate plasmid DNA. The other insertion pathways with potential applications in humans include (1) bioballistics, which requires further technical enhancement for use in humans (Fynan et al., 1993; Brouillette et al., 2016); (2) intradermal needleless administration of the DNA plasmid: in tests conducted with non-human primates, these intradermal needleless devices did not increase immunogenicity compared to conventional syringes (Rao et al., 2006); (3) intradermal tattoo, in which the DNA plasmid is delivered to the epidermal layer using thousands of injections; and (4) through the mucosa, which simulates the entry of pathogens by this route. One approach for this last route uses bacteria as carriers of a DNA plasmid to target specific cells and activate receptors of pathogen-associated molecular patterns (Becker et al., 2008). These vaccines are still under clinical testing, and microbiological research efforts have focused on HIV, hepatitis B, hepatitis C, influenza, and HPV.

Recombinant (gene) vaccines are prepared from viruses engineered to carry genes encoding antigens from other disease-causing viruses for expression in the host after inoculation. This expression induces antibody production and immunization. The immunity induced by recombinant vaccines is usually attributed to the ability of the recombinant virus to express the gene of interest at high levels within the host cells. The viral vectors used for this purpose are attenuated to the host and are therefore intrinsically safe. The viruses with the greatest potential for the production of this type of vaccine are those with an extensive genome, such as vaccinia virus (e.g., He et al., 2000). In the manufacture of such vaccines, all genes that are not essential for replication are first eliminated from the virus. Subsequently, the genes of the other virus are introduced. Recombinant vaccines are not yet available clinically but are quite promising.

Recently, the incorporation of biotechnological techniques has allowed wide access to numerous monoclonal antibodies (mAbs). Human virus-neutralizing MAbs have already been isolated from non-immune and immune sources using a range of newly developed antibody isolation technologies. One such technology employs microorganisms, such as phages, yeasts, bacteria, and viruses, to display repertoires of single-chain variable-domain antibody fragments (ScFvs), antigen-binding fragments (Fab), or domain antibodies (Dabs) on their surfaces (Carter, 2006). These antibodies can also be obtained directly from memory B cells of viral-infected patients or even from mouse lymphocyte cells (Marasco and Sui, 2007). These antibodies have also been used for the treatment of infectious diseases. Recently, two antibodies were approved for this purpose: palivizumab, a human respiratory syncytial virus (RSV)-neutralizing monoclonal antibody that blocks virus replication; and raxibacumab, which prevents binding of the protective antigen of the anthrax toxin to its receptors in host cells (The IMpact-RSV Study Group, 1998; Kummerfeldt, 2014; Baldo, 2016). MAbs represent one of the largest classes of drugs in development, and between 2010 and 2014, 17 of the 54 protein drugs approved were mAbs (31.5%). These drugs therefore provide a new and promising way of thinking about the treatment of diseases caused by microorganisms.

In addition to increasing progress in the production of vaccines and MAbs, technological advances have also increased the availability of new drugs, such as antibiotics and hormones. The boom of antibiotic discovery occurred between 1950 and 1960. However, despite the need for new antibiotics, only two new classes of antibiotics have been introduced in medicine since 1963, both of which are based on nalidixic acid (Brito and Cordeiro, 2012). Limited research results, inappropriate prescription of antibiotics, and misuse of antibiotics by the general population have threatened antibiotic potency and increased the occurrence of superbugs, i.e., microorganisms that appear at an alarming rate and are resistant to most or all clinical antibiotics in use (Srivastava et al., 2011).

In the United States, the Centers for Disease Control and Prevention estimate that antibiotic-resistant bacteria infect more than two million people annually (Nizet, 2015). In this country alone, methicillin-resistant *Staphylococcus aureus* (MRSA) accounts for approximately 10,000 cases of hospital-acquired bloodstream infections, whereas *Clostridium difficile*, associated with diarrhea, is the most common infection in the United States, with more than 80,000 estimated annual cases (Magill et al., 2014). Many multi-antibiotic-resistant gram-negative bacilli also fit the description of superbugs, such as *P. aeruginosa, K. pneumoniae, E. coli, Acinetobacter baumannii*, and *Stenotrophomonas maltophilia*, and polymyxins have emerged as the major last-line of defense against these gram-negative superbugs (Velkov et al., 2016). Recently, Ling et al. (2015) developed an important method that allowed the *in situ* growth of soil microorganisms not cultivable under laboratory conditions. Hence, chemicals produced naturally by the microorganisms could be tested, such as teixobactin, the first compound of a new important class of antibiotics. Teixobactin is capable of eliminating MRSA, and bacteria are believed not to be susceptible to developing resistance to teixobactin, which targets the lipids essential for the maintenance of the bacterial cell wall (Borghesi and Stronati, 2015). Thus, biotechnological alternatives have been developed to circumvent the problem of the low rate of discovery of new antibiotic molecules. Selenium nanoparticles (SeNPs) combined with the synergistic properties of quercetin and acetylcholine showed inhibitory effect on MRSA. A study showed that SeNPs attach to the bacterial cell wall, causing irreversible damage to the membrane, thus achieving a remarkable synergistic antibacterial effect that inhibits MRSA (Huang X. et al., 2016).

The use of recombinant microbial cells has allowed large-scale production of a large number of products of pharmaceutical interest, such as hormones, anticoagulants, high-value proteins, antibodies or antigens, and others. This has been crucial in determining the structure-function relation of proteins, as well as for developing a better understanding of immune system reactions, cell biology, and signaling events. The major microorganisms explored as biofactories are the bacterium *E. coli*, followed by the yeast *S. cerevisiae*; both prokaryotic and eukaryotic systems are constantly evolving and competing to improve their properties and intensify as platforms of choice for the production of biopharmaceuticals (Chumnanpuen et al., 2016; Sanchez-Garcia et al., 2016). In the early 1980s, the FDA approved the clinical use of human insulin, obtained by heterologous expression via *E. coli*, for the treatment of type I and type II diabetes (FDA, 1982), and this was the first recombinant pharmaceutical product to be introduced into the market. Since then, the improvement of new heterologous protein production systems via *E. coli* enabled the commercial approval of several other products, including hormones (calcitonin, parathyroid hormone, human growth hormone, glucagon, and somatropin), interferons, and interleukins (Ferrer-Miralles et al., 2009). For example, approximately 30% of commercially available recombinant proteins are currently produced in prokaryotic systems (Overton, 2014). This production method is due to the unusual physiology of the cells as well as the ease of genetically manipulating them, but the understanding is that it is possible, by adding heterologous reactions, to synthesize 1,777 non-native products from *E. coli*, of which 279 have commercial applications. Among the latter are 4-hydroxybenzoate, tyrosine, and phenylalanine, which are precursors common to a large number of non-native commercial products (Zhang et al., 2016).

By contrast, the products of heterologous proteins obtained from *S. cerevisiae* comprise hormones (insulin, insulin analogs, and glucagon), vaccines (hepatitis B virus surface antigen), and virus-like particles (VLPs) (Ferrer-Miralles et al., 2009). Prokaryotic production systems are required whenever the recombinant proteins are smaller or do not require post-translational modifications (PTMs), such as glycosylation, phosphorylation, or proteolytic cleavage. However, production in yeasts, such as *S. cerevisiae* and *Pichia pastoris*, is generally required when the target protein cannot be produced in a soluble form in a prokaryotic system, when it is rich in disulfide bonds, or when a specific PTM, essential for the biological activity of the protein, cannot be artificially created from the purified product (Jenkins, 2007; Demain and Vaishnav, 2009).

Modified strains of *S. carnosus, Corynebacterium glutamicum, B. subtilis*, and *Lactococcus lactis*, for example, have been used in the controlled biological synthesis of calcitonin, amino acids (glutamate and lysine), proinsulin (Olmos-Soto and Contreras-Flores, 2003; Sandgathe et al., 2003; Liu et al., 2016), and protein nanoparticles (Cano-Garrido et al., 2016), respectively. Further manipulations of these species are expected to create strains capable of producing a wide variety of non-native commercial products.

Filamentous fungi, used for centuries in traditional Chinese medicine, have also been evaluated for the potential production of biopharmaceuticals. Polysaccharides of secondary metabolites can be obtained using *Ganoderma lucidum, Cordyceps sinensis*, and *C. militaris* (Paterson, 2006, 2008; Wadt et al., 2015). Endophytic fungi such as *Metarhizium anisopliae* and *C. gloeosporioides* (Gangadevi and Muthumary, 2008; Liu et al., 2009) were found to be efficient in the synthesis of Taxol, being a viable alternative to obtain this antineoplastic. The great diversity of molecules produced by filamentous fungi justifies the exploration of these microorganisms, and therefore, the development of production systems in bioreactors has been encouraged.

The metabolic engineering of molecules from microorganisms has been stimulated mainly by the need for new functional biomaterials in emerging drugs (nanostructured or not) (Vázquez and Villaverde, 2013). A large number of substances with nanomedical application have emerged, including polymers, metallic nanoparticles, magnetic nanoparticles, VLPs, virions or virion components, and a growing diversity of self-organized protein materials, some with adjustable biomechanical properties such as stiffness, elasticity, adhesion or controllable disintegration or release of incorporated functional blocks or conventional chemical drugs (Rodriguez-Carmona and Villaverde, 2010). Obviously, the success of the use of microbial nanoparticles in nanotechnology and nanomedicine depends on the identification of new species of microorganisms and on the knowledge of the microbial interactions that occur in natural environments that can lead to the discovery of new molecules (Bajaj et al., 2012; Avendaño et al., 2016; Borghese et al., 2016).

Recently, the term ‘biobetter’ has been used to refer to next-generation therapeutic macromolecules, which have a more effective drug delivery system. These macromolecules are modified by chemical and/or engineering methods using molecular biology techniques to display better pharmacological properties, such as higher activity, greater stability, fewer side effects, and lower immunogenicity (Beck, 2011; Jozala et al., 2016). Because they require original research and development, and because they propose alternative methods of administration such as dermatological applications and inhaled formulations in order to minimize biological instability, biobetters still have significantly higher costs compared to reference biopharmaceutical versions (Mitragotri et al., 2014; Sandeep et al., 2016). However, the future popularization of protein engineering techniques, especially site-directed mutagenesis (SDM), which allows the substitution, elimination or insertion of one or more amino acids in the sequence of a protein, is expected to enable the availability of less expensive biobetters, which are the main growing class of biopharmaceuticals (Courtois et al., 2016; Jozala et al., 2016).

## Materials Technological Microbiology

The application of biotechnological techniques to microbiology has also made it possible to obtain a great diversity of biomaterials and biosensors. Biomaterials are artificial or natural products, usually synthesized by microorganisms in different environmental conditions, that can act in biological systems (tissues or organs). Biosensors integrate microorganisms with a physical transducer to generate a measurable signal proportional to the concentration of analytes, allowing rapid and accurate detection of analysis targets in diverse fields such as medicine, environmental monitoring, food processing, and others (D’Souza, 2001; Paitan et al., 2003; Lei et al., 2006; Su et al., 2011).

An important family of biomaterials includes the bioplastics. Bioplastics are polyesters that accumulate intracellularly in microorganisms in the form of storage granules, with physicochemical properties similar to petrochemical plastics. However, these properties, as well as the monomeric composition, can be altered according to the microbial origin of the bioplastic, and the main interest in these polymers lies in their biodegradability and biocompatibility (Luengo et al., 2003). Bioplastic can also be produced as a byproduct of biorefinery using acidogenic fermentation or pyrolysis of lignocellulosic biomass, as well as a by-product of the biotreatment of solid or liquid wastes (Ivanov and Stabnikov, 2016).

Bioplastics are being used in the manufacture of high-value-added medical materials, such as films that function as vehicles for drug delivery (Awadhiya et al., 2016). For use as medical materials, the high purity of PHAs is enhanced by using bioceramics and/or bioactive glasses that improve their biomechanical properties and increase their bioactivity (Radecka et al., 2016). Bioplastic formulations were recently tested for seed coating of agronomic species. These coatings, which contain spores of growth promoters such as *T. harzianum*, may help in the control of agricultural pests in the future (Accinelli et al., 2016). Bioplastics also have the potential to lead to the rise, within civil construction, of materials that have low incorporated energy, contributing to energy efficiency (Ivanov and Stabnikov, 2016).

Polysaccharides of microbial origin, such as chitosan, alginate, xanthan gum, and cellulose, are another class of biomaterials that have gained considerable interest for medical use because of their properties, including that they are renewable, biodegradable, and mimic the components of the extracellular matrix, which make them key elements in biological processes (Pires and Moraes, 2015; Pires et al., 2015). Chitosan can be easily recovered from the cell wall of fungi such as *A. niger, Rhizopus oryzae, Cunninghamella elegans*, and *Mucor indicus*, among others (Pochanavanich and Suntornsuk, 2002; Franco et al., 2004; Ruholahi et al., 2016; Abdel-Gawad et al., 2017). Specific interactions with extracellular matrix components allow the use of chitosan in the field of tissue engineering to repair skin, bone, and cartilage (Khor and Lim, 2003). The potential of fungal chitosan, when present in bioactive filters, to chelate heavy metals and inhibit pathogenic microbial agents in contaminated water was recently evaluated (e.g., Ruholahi et al., 2016; Tayel et al., 2016a) for the development of a renewable, ecofriendly, and cost-effective polymer that can help overcome the current problems of chemical and microbial water pollution (Tayel et al., 2016b).

Alginate is a polysaccharide synthesized by several genera of brown algae and two genera of bacteria: *Pseudomonas* and *Azotobacter* (Hay et al., 2013; Maleki et al., 2016). Its properties have broadened its use in the encapsulation or controlled release of drugs, enzymes, or cells, or as a matrix for tissue engineering (Andersen et al., 2012; Lee and Mooney, 2012), similarly to chitosan. When mixed in the aqueous phase, alginate and chitosan combine spontaneously by strong electrostatic attraction, forming a polyelectrolyte complex (PEC) that may be employed for the production of thin, transparent membranes that allow good absorption of physiological fluids, as well as the incorporation of several bioactive compounds (Pires and Moraes, 2015). Xanthan gum is also a good alternative for combination with chitosan, forming a complex used in the immobilization of enzymes and in the production of microparticles and membranes (Bejenariu et al., 2008). This exopolysaccharide is commercially synthesized by the bacterium *Xanthomonas campestris*, using different carbon sources (Barua et al., 2016; Li et al., 2016; Velu et al., 2016).

Cellulose synthesized (in abundance) by bacteria such as *Gluconacetobacter xylinus* (e.g., Huang C. et al., 2016) displays the same polymeric structure of cellulose from plants but is superior in its mechanical properties, purity, and uniformity, allowing the production of higher-quality devices (Pires et al., 2015). These devices include dialysis membranes and scaffolds for tissue engineering (Svensson et al., 2005). Microbial cellulose has great potential for the treatment of skin lesions and replacement of small-diameter blood vessels (Czaja et al., 2006).

The focus on microorganisms as an alternative in the production of biosensors is mainly due to the ability to produce them massively through cell culture (Su et al., 2011). In addition, the recombinant DNA technique has facilitated the availability of microbial biosensors in the market, providing a new direction to manipulate their selectivity and sensitivity at the DNA level. This technique consists of the construction of recombinant microbial strains that contain a reporter gene (*lux*, GFP, or *lac*Z), i.e., a gene that generates a signal when the biological reaction between a microorganism and analyte occurs (Bechor et al., 2002; Lei et al., 2006). An example of a microbial biosensor currently being used for pollutant monitoring purposes consists of immobilized recombinant *E. coli* cells expressing organophosphorus hydrolase (OPH). The OPH catalyzes the hydrolysis of organophosphorus pesticides releasing protons, whose concentration is proportional to the amount of substrate analyzed (Mulchandani et al., 1998; Kim H. J. et al., 2016). Recently, *E. coli* cells were developed to function as CadC-T7 biosensors, which are based on synthetic genetic circuits that combine a fluorescence reporter gene and heavy metal-responsive proteins. These biosensors showed high specificity for the detection of heavy metals (Kim K. R. et al., 2016). For environmental monitoring of cadmium, a biosensor in which cells express β-galactosidase in the presence of this metal is also available (Shin, 2016). Bioluminescent *E. coli* have also been used to signal DNA damage, superoxide radical production, and membrane damage caused by potentially toxic liquids (Bharadwaj et al., 2017).

In addition to the use of *E. coli* in biosensors, other microorganisms have already been evaluated. *P. putida* has already been tested as a biosensor for catechol, nitrophenol, benzene, toluene, and others (Rasinger et al., 2005; Timur et al., 2007; Banik et al., 2008); *S. cerevisiae*, for Cu^2++^ (Tag et al., 2007); *Acidithiobacillus ferrooxidans* and *Leptospirillum ferrooxidans*, for Fe^2+^, S_2_O_3_^2-^, Cr_2_O_7_^2-^, and others (Zlatev et al., 2006; Stoytcheva et al., 2009); and *Gluconobacter oxydans*, for propanediol and ethanol (Katrlik et al., 2007; Valach et al., 2009), among others. Despite the great leap forward made by biotechnology in the area of microbial biosensor development, many challenges still need to be overcome. New microorganisms still need to be evaluated for efficiency, more precise methods for immobilizing microbial cells still need to be developed, and the induction techniques need to be continuously evaluated because they may vary in terms of their efficiency depending on the analyte.

## Other Considerations

**FIGURE 5:**
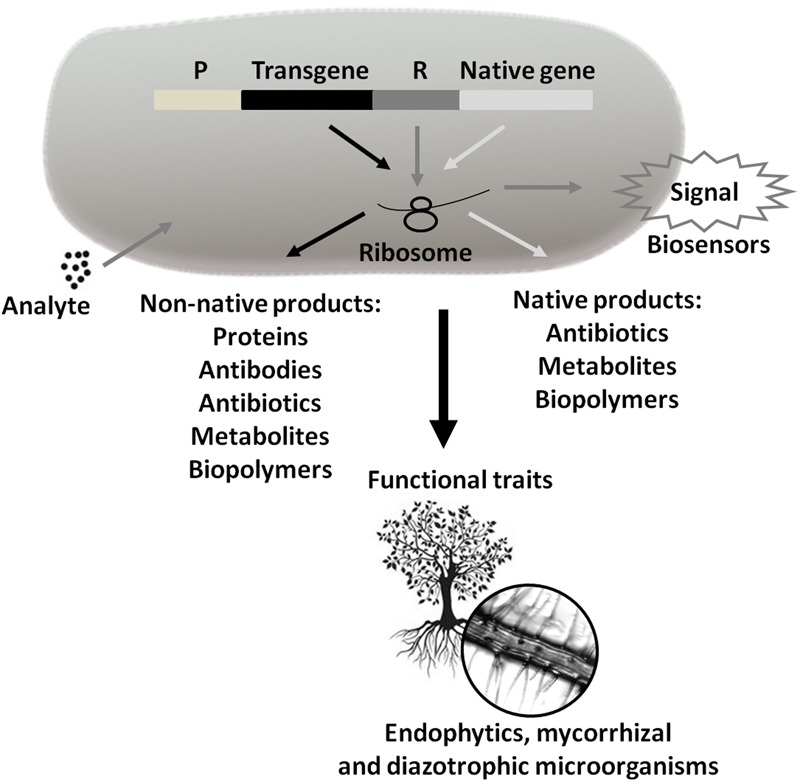
**Use of Technological Microbiology in the generation of products and services.** These products and services can be obtained from the expression of transgenes or native microbial genes (P = promoter and R = reporter). Marker expression generates signals that may indicate the presence and concentration of analytes (biosensors). In turn, the symbiotic interaction between plant species and endophytic, mycorrhizal, and/or diazotrophic microorganisms can help plant growth and development through N_2_ uptake, immobilized phosphate solubilization, siderophore production, competition with phytopathogenic species, etc. Arrows of the same color inside the bacterium signal the same pathway.

Microorganisms are the most biodiverse class, leading us to believe that the emergence and spread of new human and/or agricultural pathogens may shift from a current critical situation, aggravated by globalization, to a recurrent situation at various points in the future. Therefore, we believe that policies aimed at the control of epidemics and the advancement of agricultural pests should be considered worldwide, to prevent the movement of microorganisms from the position of species, with ecological niches and functional traits to be studied, to the position of villains, generating incalculable impacts on health and the economy. We must face the resurgence of diseases such as Zika and the appearance of superbugs as public health alerts, requiring emergency decision-making. These decisions must be made while considering the entire technological framework currently available, including transgenics and recombinant DNA, such that incentives exist so that microbiology research can be converted into products and services for society (**Figure 5**).

Not only biodiversity but also the unique nature and the biosynthetic capacities under specific environmental conditions make microorganisms the probable candidates to solve particularly complex environmental problems, such as the biodegradation of xenobiotics, or even recurrent problems, such as the decomposition of garbage and waste piles produced daily in urban environments. Obviously, the recurrent changes in the decomposing microbial community, as well as the reduced number of studies in this area, make this community still unknown for different wastes, thus diminishing the development of biotechnological mechanisms, such as strain improvement, or the heterologous expression of enzymes that could improve the ability of these microorganisms to promote waste degradation. This is a challenge to be faced by Technological Microbiology in the coming years.

The group’s biodiversity, however, also means microorganisms offer the greatest potential for the exploration of molecules and processes, and the knowledge of unconventional species, especially within the Archaea group, has stimulated the research of genes of interest. These new genes may be incorporated by recombinant technology into biologically known species, such as *E. coli* and *S. cerevisiae*, for the large-scale synthesis of products. To date, molecular strategies have advanced by establishing heterologous expression systems for the production of valuable industrial compounds, such as biofuels, chemicals, pharmaceuticals, enzymes, and food ingredients. However, Technological Microbiology has obstacles to overcome, and these obstacles extend beyond the continuous search for unconventional microbial species with valuable metabolic properties. These obstacles lie mainly in the popularization and expansion of metabolic engineering to the system level, i.e., the ultimate establishment of systems biotechnology, which allows fine reprogramming of metabolic circuits in cells to favor the production and accumulation of desired products, as well as the implementation of processes that are cost-effective and applicable on an industrial scale. Most of the time, the products and processes generated by systems biotechnology are expensive and of little benefit when implemented on a large scale. Thus, only in-depth research in this area could result in more complex and efficient microbial factories.

## Author Contributions

LV conceived this article, reviewed the existing literature, participated in writing, and created the figures. LB participated in writing and critically reviewed all content.

## Conflict of Interest Statement

The authors declare that the research was conducted in the absence of any commercial or financial relationships that could be construed as a potential conflict of interest.
